# Supplementary material for: Exogenous corticosterone and melanin-based coloration explain variation in juvenile dispersal behaviour in the barn owl (Tyto alba)
Source: PLoS One. 2021 Sep 7;16(9):e0256038. doi: 10.1371/journal.pone.0256038 (PMC8423310; doi:10.1371/journal.pone.0256038)
Supplement: Attachment — Submitted filename: Response to Reviewers.docx [file pone.0256038.s001.docx]

*Dear Cheryl S. Rosenfeld*

*Thank you for giving us the opportunity to resubmit our manuscript. Below you find our answers to the questions raised by the reviewer.*

*Sincerely, on behalf of the co-authors,*

*Bettina Almasi*

Reviewer #1: I thank the authors for sharing an engaging, well-written manuscript detailing the factors that relate to juvenile dispersal in barn owls. In particular, the finding that a short-term physiological elevation of corticosterone delayed juvenile dispersal while ultimately leading to longer final dispersal distances in females and reducing survival is very interesting and makes an important contribution to this field. I have just a few questions/comments to help improve the paper, mostly about methods and analysis.

Re. timing of implants: I think the text indicates this on lines 107-109, but the implants were put in at an age of 3 weeks, correct?

***Our response:*** *the nestlings received the implants at a mean age of 29 days; details are listed on line 116ff.*

At what age were the radio transmitters attached?

***Our response:*** *Indeed, this information is missing. We added the following on line 132ff.:* We attached the transmitters with a leg-loop harness (rubber band) on the back (Naef-Daenzer 2007) ***when nestlings were on average 49 days old (cort-juveniles: 49.6 days (range 43 – 59), placebo-juveniles: 49.3 days (41 – 59)).***

Did the measurement of plumage traits and tarsus length, etc. a few days before fledging carry any risk of force fledging the nestlings?

***Our response:*** *There is no risk of force fledging if the nestlings are younger than 55 days of age (personal experience). However, at this visit to the nest boxes we will close the nest entrance with a net for the rare cases when nevertheless a nestling tries to jump out of the nest boxes or when the oldest nestlings are older than 55 days. Before putting the nestlings back to the nest box we close the nest entrance with a sponge attached to a long rope. A few minutes after the nestlings have been set back to the nest box we pull the rope and observe the entrance for a while. It never happens that any juvenile jumps/flies out. We added the following explanation on line 133ff:* ***At this age juveniles were not yet able to fly which we confirmed by checking whether the transmitter signals are still coming from inside the nest boxes the day following the transmitter attachment. None of the juveniles had left the nest at this age.***

In addition, elsewhere another study is cited that mentions that these same nestlings underwent a stress series later during the nestling period – would it be possible to provide a timeline or other clear description in one place of the number of times that individuals were handled and/or sampled during the nestling period? Where the ages of handling/sampling the same in 2004 and 2005?

***Our response:*** *We added the following information on line 108:* ***In both years, the nest boxes were controlled regularly to monitoring the growth of the juveniles. The controls took place around hatching, when the oldest nestling reached 3 weeks, at age 25-30*, age 28-32*, age 32-36*, age 41-43 and age 50* of the oldest nestling. During these controls the nestlings were measured and weighed and where indicated with an * blood samples to measure baseline and stress-induced corticosterone were taken (for details see*** ***Almasi et al. 2012, Almasi et al. 2009).***

For the individuals that were found dead, was a cause of death apparent or discernable? This seems especially important given the difference between CORT and placebo-treated birds.

***Our response:*** *We added the following information in the result section on line 280ff.:* ***Five juveniles died after collision with a car or power line (2 cort-, and 3 placebo individuals), one placebo-individual died in a slurry-tank, one cort-individual in a ventilation shaft. From then we only found remains (feathers, carcasses) and could not determine the cause of death.***

And finally, I noted that there were some differences between the 2 years of sampling (smaller sample sizes in 2005, different numbers of birds treated as nestlings in each nest each year, as well as different frequencies of radio tracking post-fledging). However, year is not included in any of the statistical models as far as I can tell – given these differences in experimental design, I’m a bit concerned that the results may have differed between years, because of year effects or because of design differences. I’d suggest incorporating some kind of analysis that considers year/design effects.

***Our response:*** *Thank you of pointing this out. We now added the variable ‘Birth year’ in all analysis which included data of both years. Please note that we never detected any significant effect of ‘birth year’ and the results did not change whether we included birth year or not.*

**Funding Statement**

The Swiss National Science Foundation supported financially the study (no 3100A0-
104134 to LJ and no PPOOAO-102913 to AR).

CM was supported by a grant from the Swiss Confederation (no 2015.0788).
